# Surface-Dependent Hydrogen Evolution Activity of Copper Foil

**DOI:** 10.3390/ma16051777

**Published:** 2023-02-21

**Authors:** Ling-Jie Kong, Xin-Zhuo Hu, Chuan-Qi Chen, Sergei A. Kulinich, Xi-Wen Du

**Affiliations:** 1 Hefei New-Materials Institute Co., Ltd., Hefei 238200, China; 2 Institute of New Energy Materials, School of Materials Science and Engineering, Tianjin University, Tianjin 300072, China; 3 Research Institute of Science & Technology, Tokai University, Hiratsuka 259-1292, Kanagawa, Japan

**Keywords:** crystal planes control, temperature gradient annealing, copper foils, hydrogen evolution

## Abstract

Single-crystal planes are ideal platforms for catalytic research. In this work, rolled copper foils with predominantly (220) planes were used as the starting material. By using temperature gradient annealing, which caused grain recrystallization in the foils, they were transformed to those with (200) planes. In acidic solution, the overpotential of such a foil (10 mA cm^−2^) was found to be 136 mV lower than that of a similar rolled copper foil. The calculation results show that hollow sites formed on the (200) plane have the highest hydrogen adsorption energy and are active centers for hydrogen evolution. Thus, this work clarifies the catalytic activity of specific sites on the copper surface and demonstrates the critical role of surface engineering in designing catalytic properties.

## 1. Introduction

Water electrolysis is a facile method of hydrogen production via a hydrogen evolution reaction (HER) [1,2,3], for which active HER catalysts are necessary to lower reaction barrier and energy consumption [4]. The most popular acidic HER catalysts, noble metals (such as platinum and palladium), adsorb hydrogen intermediate moderately, but their scarcity and high price limit their applications [5,6]. In contrast, copper is characterized by high electrical conductivity and low price, while its weak hydrogen adsorption causes poor catalytic activity for HER [7]. Therefore, copper itself is barely used for HER catalysts in pure form, but was reported to be used in alloys, compounds, or composites [8,9,10,11].

Previous works reported that the coordination number of catalytic sites varies with the exposed crystal plane of an active surface, which in turn significantly affects the adsorption, activity, and selectivity of catalysts [12,13]. Hence, many works have been conducted to explore the intrinsic relationship between the exposed plane and its catalytic performance [14,15,16]. In this regard, single-crystal catalysts with well-defined atomic arrangements were designated and synthesized as ideal models. For example, Tian et al. [17] obtained platinum nanoparticles with (730) surfaces by means of the electrochemical method. Habas and coworkers [18] prepared platinum nanocrystals with (200) surfaces using a chemical method based on reducing agents and surfactants. Using a similar approach, Liao et al. [19] synthesized gold nanocrystals with (331) surfaces. At the same time, Habas and collaborators [18] and Yang et al. [20] realized the epitaxial growth of Pd on the surface of nanocrystals and obtained core–shell structures with the palladium (200) surface. 

The surface structure was found to make a remarkable effect on methanol oxidation, oxygen reduction, and carbon dioxide reduction reactions [13,21,22]. For instance, Kuzume and coworkers [21] analyzed the interaction of platinum surfaces with adsorbed species, such as hydrogen, oxygen, OH, electrolyte anions, and oxide formations, and discussed the effect of structure sensitivity on its electrocatalytic activity. They suggested that the stepped surface had higher catalytic activity for oxygen reduction when compared with that of the substrate [21]. Hoshi et al. found that the reduction rate of CO_2_ significantly depended on the crystal orientation: Pd(100) < Pd(111) < Pd(110) [22]. The Pd(111) surface was shown to exhibit a uniquely high activity, while the (111) one is the least active surface for CO_2_ reduction among other platinum group metals (Pt, Rh, and Ir) [22]. Nevertheless, the obtained catalysts were generally nanomaterials with a large number of vertices and edges; those sites possess adsorptive and catalytic properties different from crystal planes, making it difficult to understand the critical role of individual crystal planes [20]. 

In comparison with metal nanoparticles, a bulk single-crystal metal surface is expected to expose a sole crystal plane with the same catalytic sites, which makes it suitable for investigating the intrinsic catalytic activity of such a crystal plane [23]. Schouten et al. found that the adsorption and desorption potentials of OH on Cu(111) and Cu(200) surfaces were different in the potential range of Cu_2_O formation in alkaline media, while no OH adsorption was observed in this potential range on their Cu(110) counterpart [23]. In our previous work, we obtained a bulk copper electrode with exposed high-energy (220) and (200) planes [24]. In acidic solution, such a copper catalyst showed improved HER performance, which was attributed to the enhanced hydrogen adsorption of the (220) and (200) planes with low coordination numbers. However, the catalyst has two types of crystal planes, and thus the effect of catalytic sites on each crystal plane has not yet been elucidated. 

Recently, much progress has been made on preparing and studying single-crystal copper foils [25,26,27], and annealing treatment was found to be effective in obtaining single-crystal samples. After annealing in hydrogen gas, a polycrystalline copper foil was shown to transform into a single crystal foil with an exposed (111) surface to reduce its surface energy [27,28,29]. In contrast, high-energy surfaces such as those of (224) and (200) planes can be preserved during annealing in oxygen-contained environment [26,30]. Hu and coworkers realized the complete transformation of copper foil crystal orientation from (100) to (111) by fully reducing the oxygen content and increasing the hydrogen content in the annealing environment [30]. Moreover, there is no work on the hydrogen evolution catalytic activity of single crystal copper. In the present work, industrially rolled copper foil with (220) surface was used as the starting material, and then its (220) surface was transformed into a (200) surface by temperature gradient annealing. In acidic solution, when used as a HER catalyst, the (200) plane-exposed copper foil shows obvious enhancement in performance. Theoretical calculations show that among various catalytic sites on different copper planes, the hollow site on the (200) plane is most suited for hydrogen adsorption, endowing the (200) exposed cooper foil with the best HER performance. Thus, this work demonstrates that that the adsorption energy of copper can be effectively adjusted through surface structure control, thereby improving its HER performance.

## 2. Materials and Methods

**Scheme 1:**
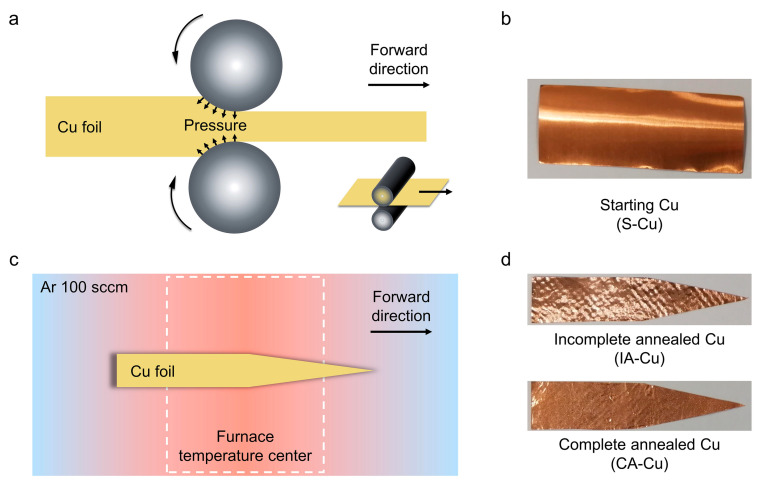
Preparation of copper foils. (**a**,**c**) are schematic diagrams of rolling process and temperature gradient annealing process, respectively; (**b**,**d**) are photographs of S-Cu, IA-Cu, and CA-Cu, respectively.

The copper material was compressed and plastically deformed between two rotating rolls into a thin foil (Scheme 1a) and then denoted as S-Cu (starting Cu). S-Cu foils were then used as raw materials for the annealing treatment (Scheme 1b). The S-Cu foil was processed into a sharp tip and put into a tube furnace filled with argon. During the annealing progress, the S-Cu foil moved through the heated area protected by 100 sccm flow of Ar gas (Scheme 1c). For comparison, two annealed samples were prepared, namely completely annealed (CA-Cu) and incompletely annealed (IA-Cu) copper foils; the former was obtained at 1100 °C and 1.95 cm min^−1^ moving speed, and the latter was prepared at 1050 °C and 19.5 cm min^−1^ moving speed (Scheme 1d). 

The morphology of the samples was observed with scanning electron microscopy (SEM, Hitachi S-4800), and their crystal structure was analyzed by means of X-ray diffractometry (XRD, Bruker D8 Advanced, with a Cu Kα radiation source). 

The HER performance was measured in a quartz cell filled with Ar-saturated 0.5 M H_2_SO_4_ for acid HER activity. A standard three-electrode system was used in electrochemical tests, for which the reference electrode was saturated calomel electrode, counter electrode was carbon rod, and the working electrodes were Cu foils. Convert all potentials with respect to the SCE to the reversible hydrogen electrode (RHE) using the equation *E* (vs. RHE) = *E* (vs. SCE) + 0.0592 pH + 0.242 V. Before electrochemical testing, 20 cycles of cyclic voltammetry (CV) were performed at a scan rate of 50 mV s^−1^ to stabilize the catalyst, and then the linear sweep voltammetry (LSV) curve was recorded at a scan rate of 5 mV s^−1^, and all polarization curves are stable after several scans. The Tafel slopes and exchange current densities were determined from the linear relationship of the overpotential to the logarithm of the current density obtained from the LSV curves. Electrochemical impedance spectroscopy (EIS) measurements were performed at a voltage of −0.613 V vs. RHE in the range of 100 kHz to 0.01 Hz. The electrochemically active surface area (ECSA) was obtained by the following Equation (1):

ECSA = *A* × DLC/*C*_*Cu*_ (1)

where *A* is the geometric area of the electrode (1 cm^2^), DLC is the double-layer capacitance of the electrode, and *C_Cu_* is the specific capacitance for smooth polycrystalline Cu, with its value being 29 µF/cm^2^ [31].

DLC was measured in the non-Faraday region. The CV cycles were carried out in 0.5 M H_2_SO_4_ solution at sweep rates of 30, 60, 90, 120, and 150 mV s^−1^. 

All DFT calculations were performed using the Vienna Ab initio Simulation Package (VASP) [32]. The projector augmented wave (PAW) [33] pseudopotential with the PBE [34] generalized gradient approximation (GGA) exchange correlation function was utilized in the computations. The cutoff energy of the plane waves basis set was 500 eV and a Monkhorst–Pack mesh of 3 × 3 × 1 was used in K-sampling. The long-range dispersion interaction was described by the DFT-D3 method. All structures were spin-polarized and all atoms were fully relaxed with the energy convergence tolerance of 10^−5^ eV per atom, and the final force on each atom was <0.05 eV Å^−1^. All periodic slabs had a vacuum layer of at least 15 Å. The bottom layers of atoms are fixed at their optimized bulk-truncated positions during geometry optimization, and the rest of the atoms could relax.

The adsorption energy of reaction intermediates can be computed using the following Equation (2):

Δ*G*_ads_ = (E_*ads_ – E_*_ – *E*_ads_) + Δ*E*_ZPE_ – TΔ*S* (2)

where ads = (*H), and (E_*ads_ – E_*_ – *E*_ads_) is the binding energy; Δ*E*_ZPE_ is the zero-point energy change, and Δ*S* is the entropy change. In this work, the values of Δ*E*_ZPE_ and Δ*S* were obtained by vibration frequency calculation.

The Gibbs free energy of the five reaction steps can be calculated by the following Equations (3) and (4):

HER

* + H^+^ + e^−^ → *H (3)

*H → 1/2 H_2_ (4)

In this work, the Gibbs free energy was calculated at *U* = 0.

## 3. Results and Discussion

### 3.1. Annealing Rolled Copper Foil into the (200) Exposed Cooper Foil

The rolled copper foil (S-Cu) was used as raw material for annealing treatment (Scheme 1a). In principle, high temperature first promotes the growth of grains at the foil tip. As the other grains in the following part move through the high-temperature zone, they are gradually annexed by the front grain, leading to a single crystal foil. The temperature and moving speed affect the recrystallization extent, so that a completely annealed copper foil (CA-Cu) can be obtained at higher temperature and lower moving speed, and incompletely annealed copper foil (IA-Cu) is prepared at lower temperatures and higher moving speeds, with their optical images being presented in Scheme 1d.

### 3.2. Characterizations of Copper Foils

**Figure 1:**
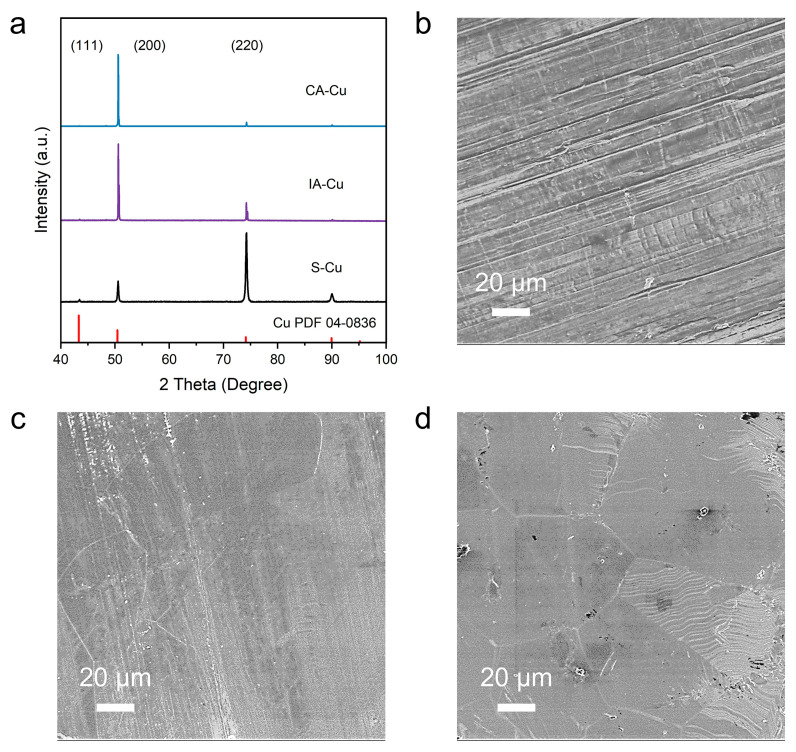
Characterization of samples S-Cu, IA-Cu, and CA-Cu. (**a**) XRD patterns and SEM images of samples S-Cu (**b**), IA-Cu (**c**), and CA-Cu (**d**).

The crystal plane orientation of copper foils was analyzed by XRD, with the obtained patterns presented in Figure 1a. The (220) peak of sample S-Cu is the strongest, indicating that the rolled copper foil is a polycrystalline copper foil with (220) as the dominated surface planes. After annealing treatment, the crystal plane orientation of copper foil changed obviously. Compared with sample S-Cu, sample IA-Cu presents a weaker (220) peak and intensified (200) peak, and its (111) peak disappeared completely. The coexistence of the (220) and (200) peaks indicates the incomplete transformation. By comparison, sample CA-Cu shows a stronger (200) peak, while its (220) peak is hardly visible. At the same time, the width of XRD peaks was also found to change after annealing. The (220) peak width of sample S-Cu became significantly larger than that of the (200) peak in samples IA-Cu and CA-Cu. According to the working principle of XRD, samples with better crystallinity have sharper and narrower diffraction peaks, while XRD of samples with fine grains typically shows broader and weaker diffraction peaks. Thus, the results of XRD analysis show that the annealed sample has better crystallinity, which proves that extensive atomic arrangements took place during the recrystallization process caused by heat treatment.

The morphology of the above samples was observed by SEM. As seen in Figure 1b, dense rolling lines can be found on the surface of sample S-Cu. After temperature gradient annealing, the surface of copper foils becomes smooth, and the rolling lines disappear. Instead, copper grains with sizes of about 200 µm appear in samples IA-Cu and CA-Cu, and the grain boundaries in sample CA-Cu are more obvious than those in sample IA-Cu (Figure 1c,d). The morphology of annealed samples observed by SEM is a direct proof of recrystallization processes and grain growth during heat treatment. The sharper grain boundaries seen in Figure 1d (sample CA-Cu) exhibit the effect of complete annealing at higher temperatures and lower moving speeds, especially in contrast with the incomplete grain boundaries seen in Figure 1c (sample IA-Cu).

### 3.3. HER Performance of Copper Foils with Different Exposed Surfaces

**Figure 2:**
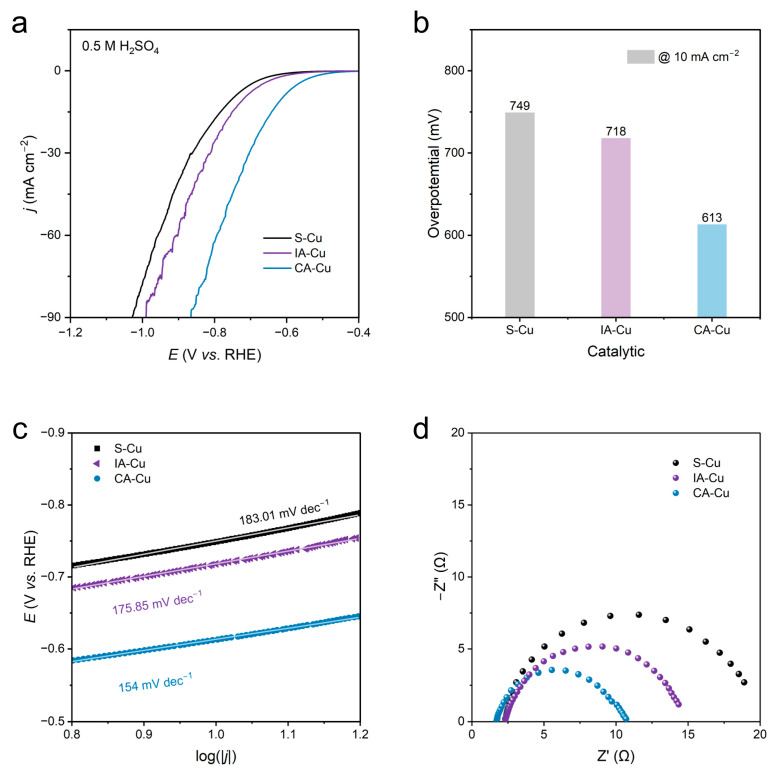
HER performance of samples S-Cu, IA-Cu, and CA-Cu. (**a**) LSV curves, (**b**) overpotentials at 10 mA cm^−2^, (**c**) Tafel curves, (**d**) Nyquist plots.

HER catalytic activities were tested in Ar-saturated 0.5 M H_2_SO_4_ solution, where the prepared copper foils were directly used as cathodes for HER. According to the linear sweep voltammetry (LSV) profiles shown in Figure 2a, sample CA-Cu exhibited better catalytic performance than its counterparts S-Cu and IA-Cu. As well seen in Figure 2b, the overpotential at 10 mA cm^−2^ of sample CA-Cu was 613 mV, which is 136 mV lower than that of sample S-Cu (749 mV) and 105 mV lower than that of sample IA-Cu (718 mV). The exchange current densities of samples S-Cu, IA-Cu, and CA-Cu were found to be 10^−3.09^, 10^−3.08^, and 10^−2.98^ mA cm^−2^, respectively, with sample CA-Cu demonstrating the highest value among the three samples. In addition, the Tafel curves were derived from LSV curves. The similar slops of the three cathodes indicate that they have similar kinetic processes, and hydrogen adsorption is the rate-determining step of their HER (Figure 2b). Sample CA-Cu presented the smallest slope (154 mV dec^−1^), and sample S-Cu presented the largest slope (183.01 mV dec^−1^), indicating that the (200) planes have the strongest hydrogen adsorption and a higher reaction rate. In the Nyquist plot (Figure 2d), sample CA-Cu exhibited the smallest semicircle, suggesting the lowest interfacial transfer resistance and the fastest electron transfer rate of the (200) planes. 

**Figure 3:**
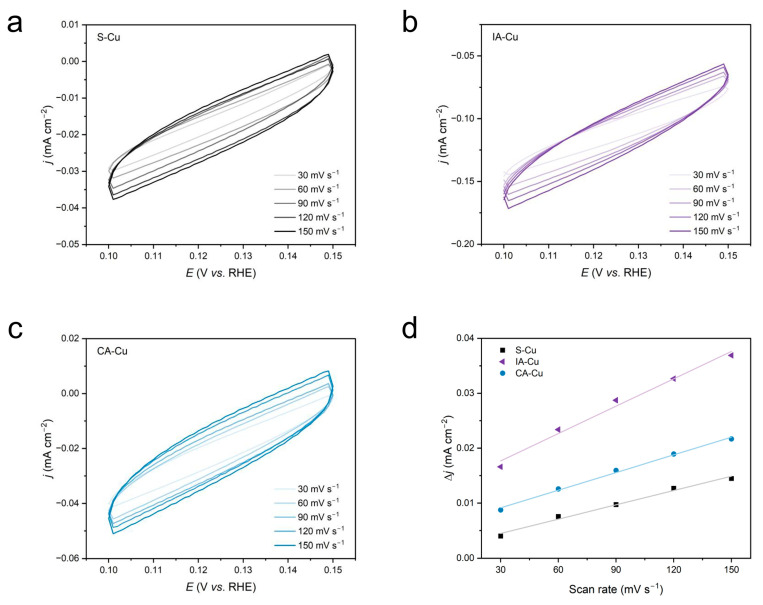
CV curves of samples S-Cu (**a**), IA-Cu (**b**), and CA-Cu (**c**). (**d**) Non-Faradaic current density (Δ*j*) as a function of scan rate (mV s^−1^) of the electrode (slopes corresponding to the DLC values).

**Figure 4:**
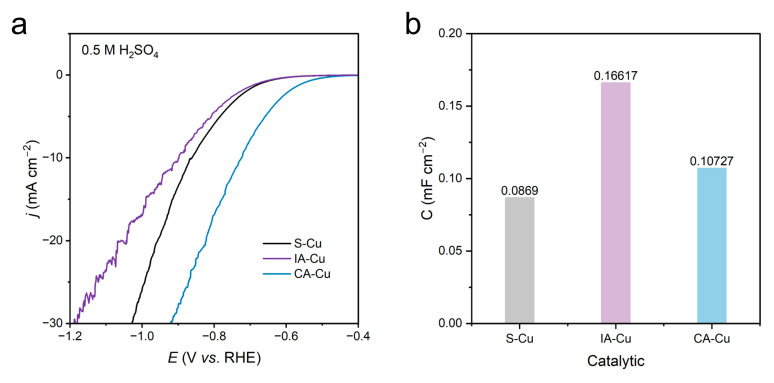
(**a**) LSV curves normalized by ECSA values, (**b**) DLC values of samples S-Cu, IA-Cu, and CA-Cu.

To explore the intrinsic activity of the foils, we obtained the electrochemically active surface area of the cathodes by measuring their double-layer capacitance (DLC) in the non-Faradaic region (Figure 3). According to the cyclic voltammetry curves in Figure 3a–c, we obtained the linear relationship between the current and scanning speed, also seen in Figure 3d. The slopes corresponding to the three samples were the double-layer capacitance (DLC), as shown in Figure 4b. The two annealed samples are seen in Figure 4b to have larger capacitance values, implying larger surface areas. Further, ECSA values of S-Cu, IA-Cu, and CA-Cu were determined as 3.0, 5.7, and 3.7 cm^2^, respectively. After being normalized to ECSA, the LSV curves corresponding to the three samples are presented in Figure 4a, where the performance of sample CA-Cu is still seen to be better than that of sample S-Cu. The results of electrochemical tests show that the hydrogen evolution activity of copper foils is related to the exposed crystal planes, and the (200) plane has the best catalytic activity.

### 3.4. DFT Calculations

**Figure 5:**
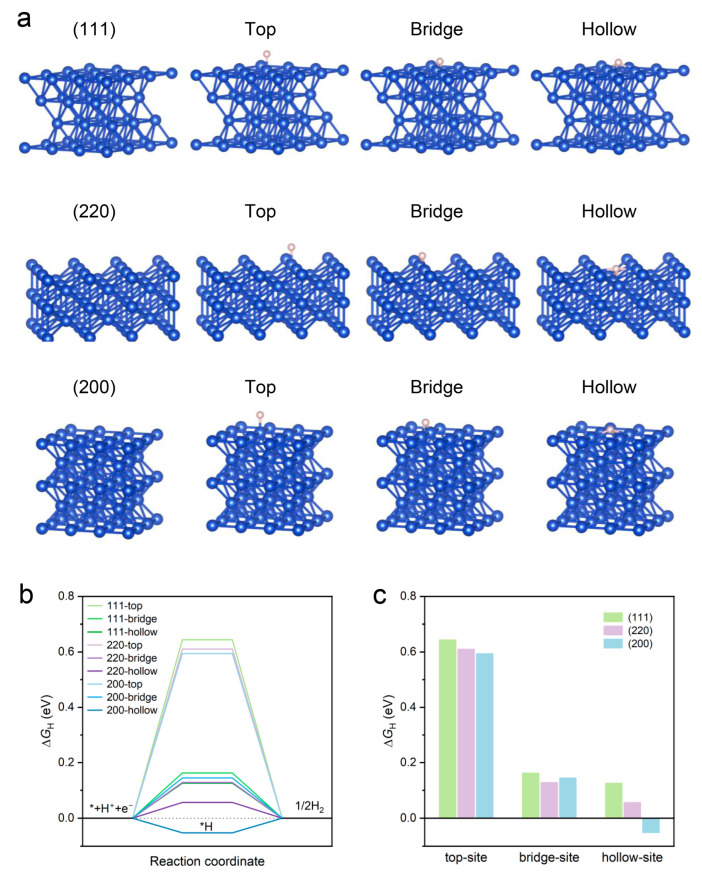
Adsorption energy of hydrogen on top, bridge, and hole sites of Cu (111), (220), and (200) planes. (**a**) Atomic models of hydrogen adsorption on Cu top, bridge, and hollow sites. (**b**) Free energy diagram, where * denotes the active site of the catalyst. (**c**) Δ*G*_H_ values for the same sites.

In order to elucidate the structure–activity relationship of hydrogen evolution over copper foils, we calculated the adsorption energy of hydrogen on (111), (110), and (100) copper planes. Moreover, there are three possible adsorptive sites on each plane, namely top, bridge, and hollow sites. Therefore, we established nine adsorption models to compare hydrogen adsorption energy on different adsorption sites (Figure 5). Figure 5a sequentially shows the atomic arrangement of (111), (110), and (100) planes of copper and the atomic model of hydrogen adsorption on Cu top, bridge, and hollow sites. It is seen in Figure 5b,c that among the nine adsorptive sites, only the hollow site on the Cu(200) plane has a negative adsorption energy, implying that this site can adsorb hydrogen intermediates effectively. Additionally, the (111) plane is always the least active surface among the three crystal planes. For top and hollow sites, the hydrogen adsorption energy of the (200) plane is superior to those of the (220) and (111) planes, consistent with the experimental results. At the same time, for bridge sites, the (200) plane is abnormally inferior to the (220) and (111) planes. In brief, the hollow site on the Cu(200) plane has the highest hydrogen adsorption energy (−0.052 eV), which is why it provides active catalytic centers for acidic HER.

## 4. Conclusions

The surface plane of copper foil was modified through temperature gradient annealing, during which the (220) surface of rolled copper foil was transformed into the (200) surface. In acidic solution, the overpotential of (200) exposed Cu foil was shown to be obviously lower than that of (220) exposed Cu foil. The experimental results and theoretical calculations show that hollow sites on (200) plane possess the best hydrogen adsorption capability among various catalytic sites on the (111), (220), and (200) copper planes. By exposing the (200) surface, the hydrogen adsorption energy of copper can be optimized, thereby improving the hydrogen evolution performance of the Cu electrode. This work elucidates the structure–activity relationship of hydrogen evolution over copper foils, and provides solid evidence for the application of surface engineering to practical HER systems. In the future, more effective techniques should be developed to expose high-index planes, aiming to provide more information on the surface-dependent hydrogen evolution activity of metallic catalysts.

## Data Availability

Not applicable.
